# Prediction of Vaginal Delivery in Pregnant Individuals with Class III Obesity Undergoing Induction of Labor: Development and Internal Validation of a Prediction Model

**DOI:** 10.3390/jcm15218371

**Published:** 2026-10-27

**Authors:** Eran Ashwal, Wan Yao Chen, Manal Massalha, Zainab Doleeb, Noora Alshamsi, Chloe Lau, Maclite Tesfaye, Cynthia Maxwell

**Affiliations:** 1 Department of Obstetrics, McMaster University, Hamilton, ON L8S 4K1, Canada; eran.ashwal@mcmaster.ca; 2 Women’s College Research Institute, Women’s College Hospital, Toronto, ON M5S 1B2, Canada; cynthiadr.maxwell@sinaihealth.ca; 3 Department of Nursing, Toronto Metropolitan University, Toronto, ON M5B 2K3, Canadam.tesfaye@mail.utoronto.ca (M.T.); 4 Ruth and Bruce Rappaport Faculty of Medicine, Technion, Israel Institute of Technology, Haifa 31096, Israel; 5 Department of Obstetrics and Gynecology, Temerty Faculty of Medicine, University of Toronto, Toronto, ON M5G 1E2, Canada; 6 Department of Obstetrics and Gynecology, Schulich School of Medicine and Dentistry, University of Western Ontario, London, ON N6A 5C1, Canada; chloehy.lau@mail.utoronto.ca; 7 Department of Obstetrics and Gynecology, Sinai Health, Mount Sinai Hospital, Toronto, ON M5G 1Z5, Canada

**Keywords:** obesity, morbid, cesarean section, labor, induced, delivery, obstetric, decision support techniques

## Abstract

**Objective:** The objective of this study was to develop and internally validate a model predicting vaginal delivery after labor induction in individuals with class III obesity, and to describe maternal and neonatal outcomes by delivery mode. **Methods:** This was a retrospective cohort study of singleton term inductions in individuals with class III obesity (BMI ≥ 40 kg/m^2^) and no prior cesarean delivery at a tertiary care center (2010–2020). Eight predictors available at admission for induction were prespecified: maternal age, parity, pre-pregnancy BMI, gestational weight gain, prior bariatric surgery, hypertensive disorders, diabetes mellitus, and cervical dilation. Missing data were addressed using multiple imputation. Internal validation was carried out using bootstrap resampling, and coefficients were shrunken using the optimism-corrected calibration slope. **Results:** Of 183 individuals, 92 (50.3%) achieved vaginal delivery. The mean BMI was 50.5 ± 5.2 kg/m^2^; 68.9% were nulliparous. Multiparity (adjusted odds ratio [aOR] 7.38, 95% CI 3.26–16.70) and younger maternal age (aOR 0.91 per year, 95% CI 0.85–0.98) were associated with vaginal delivery; BMI was not (aOR 0.99 per kg/m^2^, 95% CI 0.93–1.06). Labor dystocia accounted for 69 of 91 cesarean deliveries (75.8%). Composite maternal morbidity occurred in 9.8% of patients after vaginal delivery and in 14.3% after cesarean delivery. The optimism-corrected AUC was 0.746 (95% CI 0.675–0.806) and the optimism-corrected calibration slope was 0.74. **Conclusions:** Half of the individuals with class III obesity achieved vaginal delivery after labor induction. Parity, not BMI, was the main determinant. This internally validated model requires external validation before it is used for counseling.

## 1. Introduction

Class III obesity (body mass index [BMI] ≥ 40 kg/m^2^) is increasingly common in obstetric practice, and national data demonstrate continued increases in pre-pregnancy obesity in recent years [1]. In pregnancy, obesity is associated with increased medical complications and cesarean delivery rates, amplifying its clinical burden [2,3,4].

Labor induction is common in patients with class III obesity as obesity-related comorbidities and obstetric indications accumulate over gestation [2,3,4]. However, induction is often more complex and failure rates increase with higher BMI [5,6]. Cesarean delivery in this population can be associated with greater complications and technical difficulty [2,3,4], making delivery counseling and birth planning particularly complex for this high-risk subgroup.

Despite the frequency and risk profile of induction associated with class III obesity, there is no broadly validated prediction model specific to higher BMI categories that is clearly usable at the time labor induction is planned. The Bishop score was designed for cervical assessment rather than comprehensive delivery mode prediction [7], and national guidance on failed induction standardizes criteria but does not provide BMI-specific recommendations [8].

Given the elevated rates of induction failure and cesarean delivery in this population, identifying patients with a higher likelihood of vaginal delivery is central to counseling and clinical decision-making. We therefore aimed to (1) identify clinically available predictors of vaginal delivery among patients with class III obesity undergoing labor induction and (2) develop and internally validate a prediction model for successful vaginal delivery using these predictors.

## 2. Materials and Methods

### 2.1. Study Design and Setting

We performed a retrospective cohort study at a single academic tertiary care center providing maternal–fetal medicine services in Ontario, Canada (1 January 2010 to 31 December 2020). The Institutional Research Ethics Board approved the study with a waiver of informed consent (Protocol #19-0353-C). Methods and reporting followed TRIPOD guidance for prediction model development with internal validation (Type 1b) [9]. The completed TRIPOD checklist is provided in Supplementary Table S1.

### 2.2. Study Population

We included individuals with a singleton pregnancy, pre-pregnancy BMI ≥ 40 kg/m^2^, induction of labor at ≥37 weeks’ gestation with intent for vaginal delivery, and cephalic presentation at induction initiation. Induction was defined as any intervention used to start labor. When the cervix was unfavorable, cervical ripening was performed with a prostaglandin, a transcervical balloon catheter, or both; when it was favorable, labor was induced with amniotomy and/or oxytocin. The method was chosen by the attending obstetrician after the admission cervical examination. Oxytocin use at any point after admission was recorded. There were no standard institutional criteria for failed induction; the timing of cesarean delivery for failed induction or labor arrest was at the discretion of the attending obstetrician.

Exclusion criteria included contraindications to vaginal delivery (e.g., placenta previa, prior classical uterine incision, and prior full-thickness myomectomy), candidates for trial of labor after cesarean (TOLAC), planned cesarean delivery without labor attempt, and incomplete patient records. We excluded patients with a prior cesarean delivery because management pathways and induction options differ, and numbers of such cases were insufficient to support robust subgroup modeling.

Gestational age was determined using the best obstetric estimate per standard pregnancy dating principles [10].

For descriptive analyses, class III obesity was categorized as BMI 40.0–49.9 (class 3A), 50.0–59.9 (class 3B), and ≥60 kg/m^2^ (class 3C) [11].

### 2.3. Predictor Variables

Eight candidate predictors were prespecified based on clinical relevance, biological plausibility, the prior literature, and availability at admission for induction [12,13,14]: maternal age (years), parity (nulliparous vs. multiparous), pre-pregnancy BMI (kg/m^2^), gestational weight gain (kg; last recorded weight before admission minus early pregnancy weight), prior bariatric surgery (yes/no), cervical dilation at admission (cm at the first vaginal examination, before cervical ripening; recorded as 0 for a closed cervix), hypertensive disorder of pregnancy (HDP) (yes/no), and diabetes mellitus (pregestational or gestational; yes/no). Because individuals with a prior cesarean delivery were excluded, every multiparous individual had at least one previous vaginal birth, and parity in this cohort is equivalent to prior vaginal birth. The type of bariatric procedure was not recorded.

The model was designed for individuals in whom induction has already been planned, at admission after the first cervical examination and before cervical ripening starts. It does not address the choice between induction and planned cesarean delivery, because no individual in the cohort had a planned cesarean. Induction indication and ripening method were not entered as predictors. The ripening method is selected after the admission cervical examination and is part of the induction process rather than information available before it, and the recorded indication was often non-specific. Both are reported descriptively.

Estimated fetal weight (EFW) was not included in the primary model. Ultrasound estimates within 14 days of delivery were inconsistently available, and ultrasound EFW is less accurate in individuals with class III obesity [15]. Birth weight was used instead in a sensitivity analysis, as a best-case estimate of what fetal size could add to the model.

### 2.4. Outcome Variables

The primary outcome was vaginal delivery (spontaneous or operative).

Secondary outcomes were composite maternal morbidity (chorioamnionitis/intrapartum fever, blood transfusion, postpartum infection excluding mastitis, or readmission to hospital within 7 days) and composite neonatal morbidity (need for delivery room resuscitation, NICU admission, or 5 min Apgar score < 7). Estimated blood loss and length of postpartum stay were reported separately and were not part of the maternal composite. Shoulder dystocia was reported among vaginal deliveries only. Secondary outcomes were compared by delivery mode descriptively and without adjustment. Delivery mode is the result of labor rather than an assigned exposure, and these comparisons do not estimate the effect of cesarean delivery.

### 2.5. Missing Data

Missing data were most frequent for cervical dilation (41 of 183, 22.4%) and gestational weight gain (2 of 183); the other predictors were complete. Individuals with and without recorded cervical dilation are compared in Section 3.

Missing values were imputed by multiple imputation using chained equations with predictive mean matching (30 datasets, fixed random seed) [16]. The imputation model included all eight predictors and the outcome. Coefficients, standard errors, and *p*-values were pooled across the imputed datasets using Rubin’s rules [17]. A complete-case analysis was performed as a sensitivity analysis.

### 2.6. Statistical Analysis and Model Development

Baseline characteristics are reported descriptively, and differences between delivery modes are expressed as standardized mean differences (SMDs); *p*-values are not reported for baseline characteristics. We fit a multivariable logistic regression model with vaginal delivery as the outcome, and all prespecified predictors were entered simultaneously (full-model approach) to avoid instability from automated selection [18]. Non-linearity was assessed by adding a quadratic term for each continuous predictor, and collinearity was assessed using variance inflation factors.

### 2.7. Model Performance, Internal Validation, and Calibration

Discrimination was quantified by the area under the ROC curve (AUC). Calibration was assessed with the calibration intercept and calibration slope and displayed as a calibration plot of observed proportions in deciles of predicted probability with a smoothed calibration curve. The Hosmer–Lemeshow test was not used because of its known limitations [19].

Internal validation used 200 bootstrap samples within each imputed dataset [20]. In each sample, the model was refitted and its performance was measured in the bootstrap sample and in the original data; the mean difference (optimism) was subtracted from the apparent performance, and results were averaged across imputed datasets. The 95% CI of the optimism-corrected AUC was obtained from bootstrap percentile intervals of the apparent AUC, shifted by the estimated optimism.

To correct for overfitting, all regression coefficients were multiplied by the optimism-corrected calibration slope (uniform shrinkage), and the intercept was re-estimated so that the mean predicted probability equaled the observed rate [18,20]. The shrunken coefficients were used for the prediction equation, the worked examples, and the point score; Table 1 reports the unshrunken pooled estimates. To illustrate how the equation could be applied, it was also converted to a point score in which one point equals 0.05 on the log-odds scale, with continuous predictors grouped into clinical categories. Agreement between score-based and equation-based probabilities was checked in individuals with complete data.

**Table 1:** Multivariable logistic regression model (8 predictors), pooled across 30 imputed datasets.

Predictor	β (SE)	aOR	95% CI	*p*-Value	Shrunken β
Intercept	2.801 (2.125)			0.187	2.047
Maternal age, per year	−0.094 (0.036)	0.91	0.85–0.98	0.009	−0.069
Multiparous	1.998 (0.417)	7.38	3.26–16.70	<0.001	1.470
Pre-pregnancy BMI, per kg/m^2^	−0.007 (0.034)	0.99	0.93–1.06	0.840	−0.005
Gestational weight gain, per kg	−0.012 (0.012)	0.99	0.96–1.01	0.353	−0.008
Prior bariatric surgery	−1.654 (1.044)	0.19	0.02–1.48	0.113	−1.217
Cervical dilation at admission, per cm	0.206 (0.154)	1.23	0.91–1.66	0.181	0.152
Hypertensive disorder	−0.473 (0.356)	0.62	0.31–1.25	0.184	−0.348
Diabetes mellitus	0.278 (0.411)	1.32	0.59–2.95	0.500	0.204

**Figure 1:**
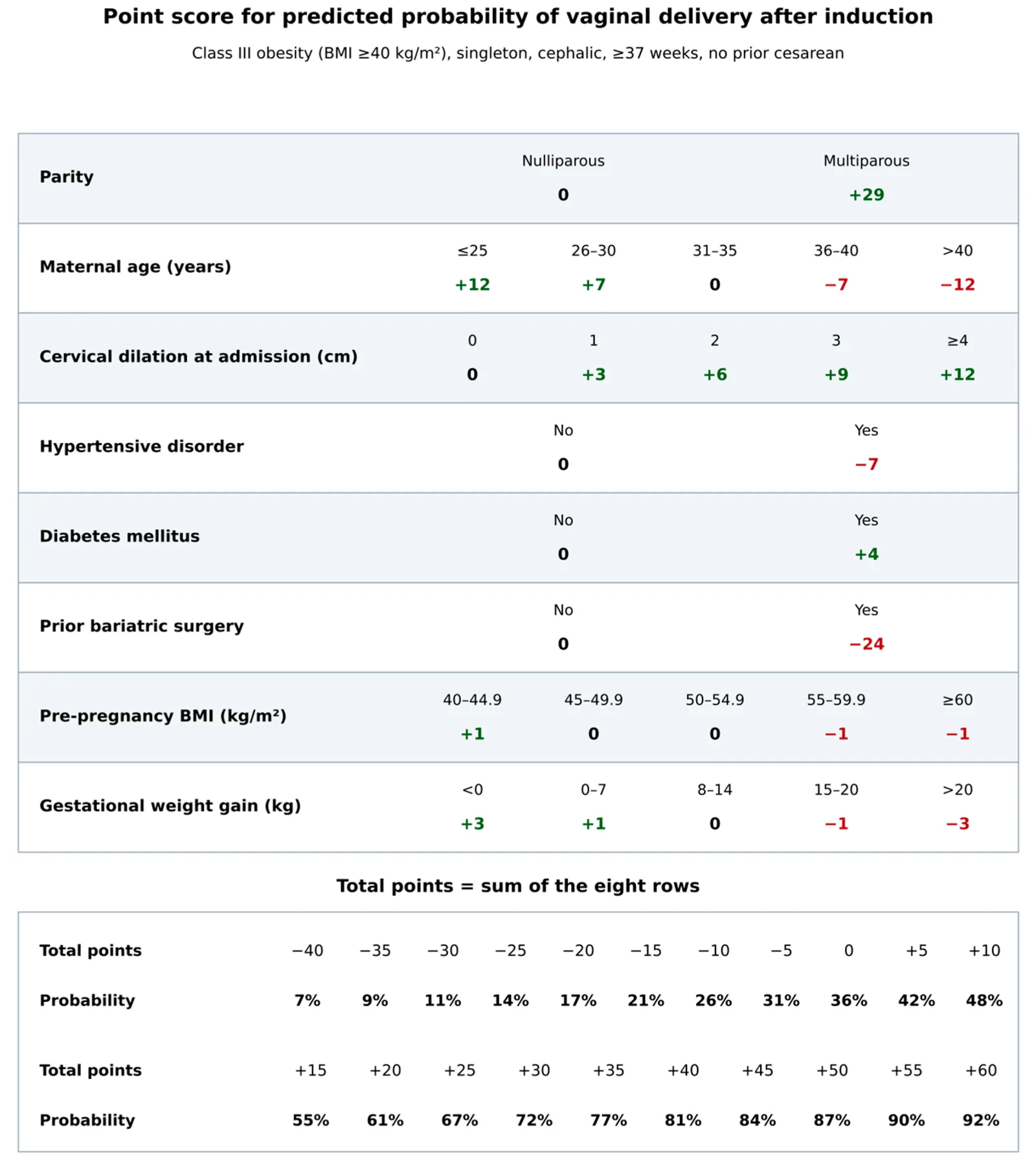
Illustrative point score for the predicted probability of vaginal delivery after induction. Points were derived from the shrunken coefficients (uniform shrinkage factor 0.74); one point equals 0.05 on the log-odds scale, and continuous predictors were grouped into the categories shown. Predicted probability = 1/(1 + exp(−(−0.56 + 0.05 × total points))). The reference profile (0 points) is a nulliparous individual aged 33 years with BMI 50 kg/m^2^, weight gain of 10 kg, closed cervix, and no hypertensive disorder, diabetes, or bariatric surgery (predicted probability 36%). In the 141 individuals with complete data, score-based probabilities differed from the exact equation by a mean of 2.2 percentage points (maximum 7.8). The score is internally validated only and is not intended for clinical use before external validation.

Coefficients, standard errors, odds ratios, 95% CIs, and *p*-values were pooled across 30 imputed datasets using Rubin’s rules. These are the unshrunken estimates. For the prediction equation (Section 4.5), Table 2, and Figure 1, all coefficients were multiplied by the optimism-corrected calibration slope (0.74), and the intercept was re-estimated. Predictors whose confidence intervals include 1 were retained for model stability and should not be interpreted as having no effect. aOR = adjusted odds ratio; CI = confidence interval; SE = standard error.

**Table 2:** Worked examples of predicted probability of vaginal delivery (illustrative only).

Scenario	Age, Years	Parity	BMI	GWG, kg	Cervical Dilation, cm	HDP	DM	Bariatric Surgery	Predicted Probability of Vaginal Delivery
Favorable multiparous	28	M	45	10	2	No	No	No	83%
Older nulliparous with HDP	38	N	52	10	0	Yes	No	No	22%
Young nulliparous, favorable cervix	25	N	48	10	3	No	No	No	61%
Multiparous after bariatric surgery	35	M	42	10	1	No	No	Yes	44%
Nulliparous, BMI ≥ 60 with HDP and DM	32	N	65	10	1	Yes	Yes	No	36%

Predicted probabilities were calculated with the shrunken equation in Section 4.5: linear predictor = 2.047 − 0.069 (age) + 1.470 (multiparous) − 0.005 (BMI) − 0.008 (GWG) − 1.217 (bariatric) + 0.152 (cervix) − 0.348 (HDP) + 0.204 (DM); probability = 1/[1 + exp (−linear predictor)]. Examples are illustrative, are not validated clinical recommendations, and assume no prior bariatric surgery unless stated. BMI = body mass index (kg/m^2^); DM = diabetes mellitus; GWG = gestational weight gain; HDP = hypertensive disorder of pregnancy; M = multiparous; N = nulliparous.

### 2.8. Clinical Utility

Decision curve analysis was used to describe the net benefit of the model across threshold probabilities of 10% to 90% [21]. Because every individual in the cohort was induced, this analysis does not compare induction with planned cesarean delivery. A positive classification was a predicted probability of vaginal delivery at or above the threshold. Net benefit was calculated as (true positives/n) − (false positives/n) × [pt/(1 − pt)], where pt is the threshold probability. The term pt/(1 − pt) is the weight given to a false-positive classification (an individual predicted to deliver vaginally who had a cesarean delivery) relative to a true positive classification. The model was compared with the scenario of assuming vaginal delivery for all and for none.

### 2.9. Sensitivity Analyses

Three sensitivity analyses were prespecified: (1) complete-case analysis; (2) addition of birth weight to the primary model, as a best-case estimate of the contribution of fetal size; and (3) an exploratory parsimonious model. The predictors of the parsimonious model (maternal age, parity, and cervical dilation) were chosen after the primary model was fitted, and this model did not replace the prespecified model. Discrimination, calibration, and coefficients were compared across specifications.

### 2.10. Sample Size

All eligible individuals in the study period were included. With 92 vaginal deliveries and 8 predictors, the events-per-variable ratio was 11.5 [22]. This meets the traditional threshold of 10 events per variable but not the criteria of Riley et al. [23], which, for 8 predictor parameters, an outcome prevalence of 50%, and the observed Cox–Snell R^2^ of 0.24, require approximately 262 individuals to keep expected shrinkage at or below 10%. Overfitting was therefore expected and was addressed by shrinkage, as described above.

### 2.11. Software

Analyses were performed in Python 3.13 using pandas, numpy, statsmodels, scikit-learn, and matplotlib.

## 3. Results

### 3.1. Study Population

Among 183 individuals with class III obesity undergoing term induction, 92 (50.3%) achieved vaginal delivery and 91 (49.7%) underwent cesarean delivery. Labor dystocia was the indication for 69 of the 91 cesarean deliveries (75.8%), and non-reassuring fetal heart rate was the indication for 19 (20.9%). Cervical dilation at cesarean was recorded in 79 cases (mean 4.6 ± 2.3 cm); 61 of these 79 (77.2%) were performed before 6 cm.

Baseline characteristics stratified by delivery mode are shown in Table 3. Compared with those who underwent cesarean delivery, individuals achieving vaginal delivery were younger (31.1 ± 4.8 vs. 33.3 ± 5.3 years; SMD 0.42), more often multiparous (50.0% vs. 12.1%; SMD 0.90), and had greater cervical dilation at admission (1.7 ± 1.4 vs. 1.0 ± 1.3 cm; SMD 0.47). Pre-pregnancy BMI was similar between groups (50.5 ± 5.3 vs. 50.5 ± 5.3 kg/m^2^; SMD 0.01). The recorded indication for induction was a hypertensive disorder in 46 individuals (25.1%), diabetes in 28 (15.3%), and other or unknown in 55 (30.1%). Cervical ripening was used in 140 individuals (76.5%): prostaglandin alone in 50, a balloon catheter alone in 56, and both in 34. The remaining 43 (23.5%) were induced with amniotomy and/or oxytocin alone, and 32 of these 43 (74.4%) delivered vaginally. Oxytocin was used in 147 of the 161 individuals with this information recorded (91.3%).

At admission, the cervix was closed in 49 of the 142 individuals with dilation recorded (34.5%) and dilated 1 cm or less in 84 (59.2%). Information on cervical dilation at admission was missing in 41 individuals (22.4%). The patients were more often nulliparous than those with a recorded value (80.5% vs. 65.5%; SMD 0.34) and less often delivered vaginally (41.5% vs. 52.8%; SMD 0.23); other characteristics were similar (SMD ≤ 0.18) (Supplementary Table S2).

### 3.2. Vaginal Delivery Rates by Subgroup

**Figure 2:**
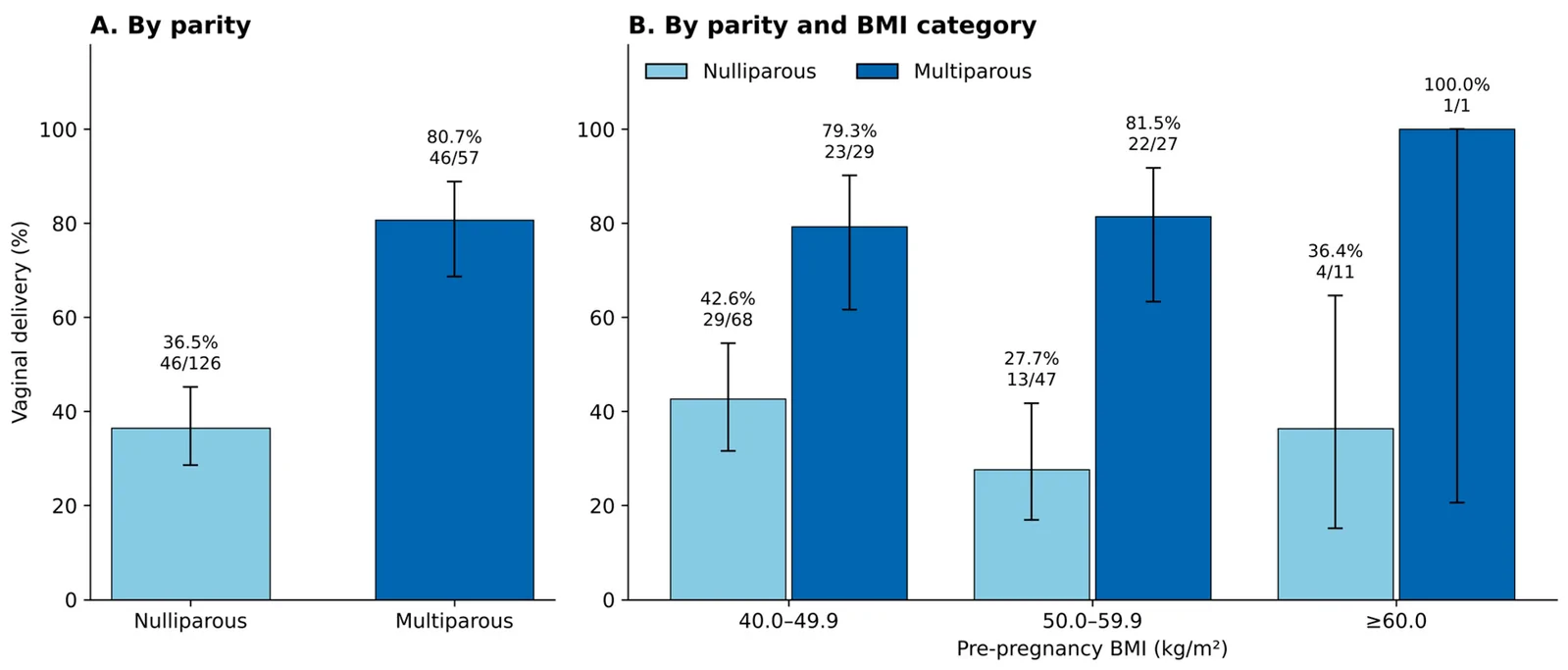
Vaginal delivery rates (**A**) by parity and (**B**) by parity within BMI category. Bars show the percentage with 95% Wilson confidence intervals; numerators and denominators are shown above each bar. The BMI ≥ 60.0 kg/m^2^ category contains 11 nulliparous individuals and one multiparous individual, and its estimates are imprecise. Subgroup comparisons are descriptive.

Vaginal delivery rates differed by parity: 80.7% (46/57; 95% CI 68.7–88.9) in multiparous and 36.5% (46/126; 95% CI 28.6–45.2) in nulliparous individuals (Figure 2). Rates were similar across BMI categories: 53.6% (52/97) for BMI 40.0–49.9, 47.3% (35/74) for 50.0–59.9, and 41.7% (5/12) for ≥60.0 kg/m^2^.

Within each BMI category, multiparous individuals had higher vaginal delivery rates than nulliparous individuals: 79.3% (23/29) vs. 42.6% (29/68) for BMI 40.0–49.9 and 81.5% (22/27) vs. 27.7% (13/47) for BMI 50.0–59.9. The BMI ≥ 60.0 category included 11 nulliparous individuals, 4 of whom (36.4%) delivered vaginally, and a single multiparous individual, so estimates in this category are imprecise. These subgroup analyses are descriptive.

Secondary outcomes are summarized in Table 4 and Supplementary Figure S1. These are unadjusted, descriptive comparisons. Composite maternal morbidity occurred in 9 of 92 individuals (9.8%) after vaginal delivery and 13 of 91 (14.3%) after cesarean delivery (risk difference of 4.5 percentage points, 95% CI −5.2 to 14.3). Composite neonatal morbidity occurred in 9 of 92 (9.8%) and 17 of 91 patients (18.7%), respectively (risk difference 8.9 percentage points, 95% CI −1.3 to 19.2; SMD 0.26), mainly because of delivery room resuscitation. The study was not powered for these comparisons, and the absence of a statistically significant difference does not indicate equivalence. Estimated blood loss was higher after cesarean delivery (839 ± 266 vs. 382 ± 234 mL; SMD 1.82), as expected for the mode of delivery; it was recorded in 25 of the 92 vaginal deliveries and 82 of the 91 cesarean deliveries, so the vaginal delivery estimate is based on a minority of the group.

## 4. Prediction Model Development

### 4.1. Multivariable Analysis

**Figure 3:**
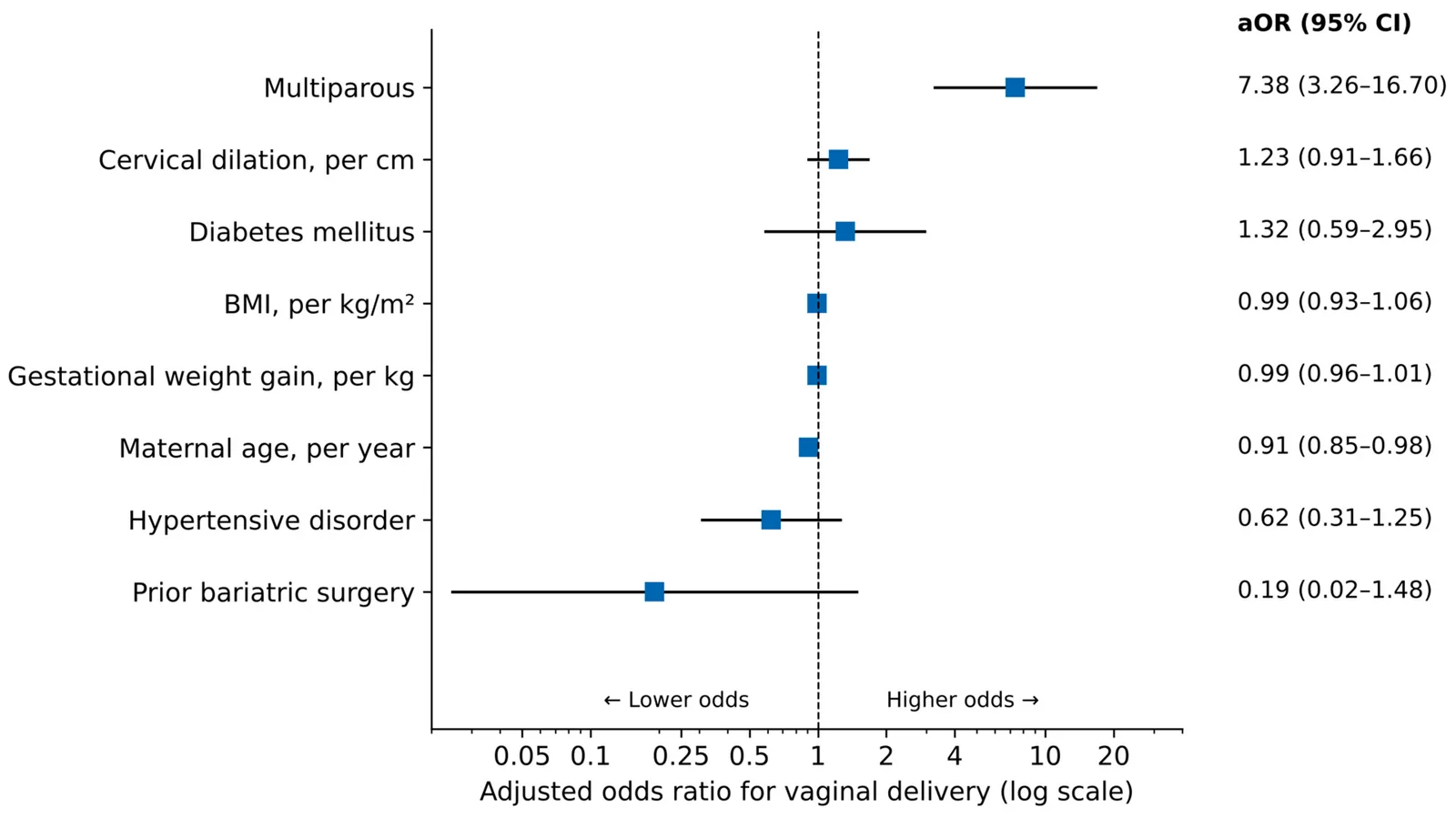
Adjusted odds ratios for vaginal delivery from the multivariable model, pooled across 30 imputed datasets using Rubin’s rules (unshrunken estimates). Horizontal lines show 95% confidence intervals; the *x*-axis is logarithmic and the dashed line marks an odds ratio of 1. All eight prespecified predictors were retained for model stability; predictors whose confidence intervals include 1 should not be interpreted as having no effect.

The multivariable logistic regression model included all eight prespecified predictors (Table 1). Multiparity was the dominant predictor (aOR 7.38; 95% CI 3.26–16.70), and maternal age was inversely associated with vaginal delivery (aOR 0.91 per year; 95% CI 0.85–0.98). The confidence intervals of the other predictors included 1, including pre-pregnancy BMI (aOR 0.99 per kg/m^2^; 95% CI 0.93–1.06). Prior bariatric surgery had a low point estimate (aOR 0.19; 95% CI 0.02–1.48) based on six individuals, and this estimate is too imprecise to interpret (Figure 3). These predictors were retained for model stability, not because they showed an effect. There was no evidence of non-linearity for the continuous predictors, and all variance inflation factors were below 1.2.

### 4.2. Model Performance

**Figure 4:**
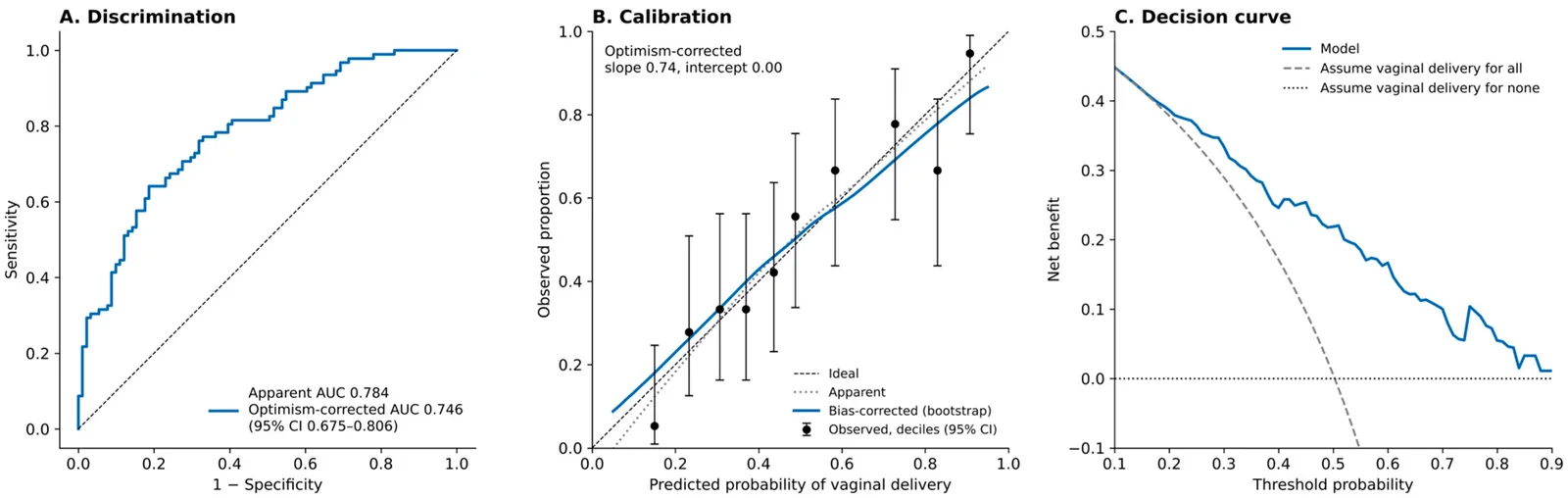
Model performance. (**A**) Receiver operating characteristic curve, drawn from predicted probabilities averaged across the imputed datasets. The apparent AUC (mean across imputed datasets) was 0.784, and the optimism-corrected AUC was 0.746 (95% CI 0.675–0.806). (**B**) Calibration: observed proportion of vaginal delivery in deciles of predicted probability (points, 95% CI), with apparent and bootstrap bias-corrected smoothed calibration curves; the dashed line represents ideal calibration. The calibration slope is 1.00 by definition in the development data; the optimism-corrected calibration slope was 0.74 and the optimism-corrected intercept was 0.00. (**C**) Decision curve for the shrunken model, compared with assuming vaginal delivery for all and for none. Net benefit = (true positives/n) − (false positives/n) × [pt/(1 − pt)], where a positive classification is a predicted probability of vaginal delivery ≥ the threshold pt. All individuals were induced; the curve does not compare induction with planned cesarean delivery.

The apparent AUC was 0.784. Bootstrap optimism was 0.038, giving an optimism-corrected AUC of 0.746 (95% CI 0.675–0.806) (Figure 4A). The Brier score was 0.190 (optimism-corrected 0.211). The calibration slope is 1.00 by definition in the development data; after bootstrap correction it was 0.74, with an intercept of 0.00 (Figure 4B). This indicates that unshrunken predictions would be too extreme in new individuals, and all coefficients were therefore multiplied by 0.74 for the prediction equation and the point score. All performance measures are listed in Supplementary Table S3.

### 4.3. Classification Performance at Prespecified Thresholds

Rather than reporting a single, sample-dependent “optimal” cut-off, we report the performance of the shrunken model at three predicted probabilities of vaginal delivery (30%, 50%, and 70%). These describe low, intermediate, and high predicted probability and are not thresholds for recommending cesarean delivery. At 30%, sensitivity for vaginal delivery was 96.7% and specificity was 28.6% (PPV 57.8%, NPV 89.7%); at 50%, sensitivity was 64.1% and specificity was 79.1% (PPV 75.6%, NPV 68.6%); and at 70%, sensitivity was 40.2% and specificity was 91.2% (PPV 82.2%, NPV 60.1%).

### 4.4. Clinical Utility

Across threshold probabilities of 10% to 90%, the model had higher net benefit than assuming vaginal delivery for none (Figure 4C). Its net benefit was similar to assuming vaginal delivery for all below a threshold of about 20%, and higher above it. This analysis describes how well the model separates individuals who delivered vaginally after induction from those who did not. It does not show that using the model to choose between induction and planned cesarean delivery would improve outcomes.

### 4.5. Illustrative Application of the Model

The shrunken prediction model is

Linear predictor = 2.047 − 0.069 (age) + 1.470 (multiparous) − 0.005 (BMI) − 0.008 (GWG) − 1.217 (bariatric surgery) + 0.152 (cervical dilation) − 0.348 (HDP) + 0.204 (DM)

Predicted probability = 1/[1 + exp (−linear predictor)]

where age is in years, multiparous has a value of 1 if multiparous and 0 if nulliparous, BMI is pre-pregnancy body mass index in kg/m^2^, GWG is gestational weight gain in kg, bariatric surgery is 1 if history of bariatric surgery and 0 otherwise, cervical dilation is in cm at admission, HDP is 1 if any hypertensive disorder of pregnancy and 0 otherwise, and DM is 1 if any diabetes mellitus (pregestational or gestational) and 0 otherwise.

Table 2 gives worked examples, which are illustrative only. Figure 2 presents a point score derived from the same equation. The score is an illustration of the equation and is not intended for clinical decision-making. In the 141 individuals with complete data, score-based probabilities differed from equation-based probabilities by a mean of 2.2 percentage points (95th percentile 5.1; maximum 7.8).

### 4.6. Sensitivity Analyses

**Birth weight:** Adding birth weight to the primary model changed the apparent AUC from 0.784 to 0.788 and left the optimism-corrected AUC unchanged (0.746 for both models). Birth weight was not associated with vaginal delivery (aOR 0.94 per 100 g; 95% CI 0.86–1.02). Coefficients for multiparity and maternal age changed by less than 7%, and the events-per-variable ratio fell from 11.5 to 10.2.

**Complete-case analysis:** The complete-case analysis included 141 individuals (77.0%); of the 142 with recorded cervical dilation, one had no recorded gestational weight gain. Discrimination was similar (apparent AUC 0.781). Multiparity remained the dominant predictor (aOR 6.64; 95% CI 2.69–16.39), BMI was not associated with delivery mode (aOR 1.02; 95% CI 0.95–1.10), and the estimate for maternal age was similar but less precise (aOR 0.94; 95% CI 0.87–1.01). Results of all sensitivity analyses are given in Supplementary Table S4.

**Parsimonious model:** The exploratory model with maternal age, parity, and cervical dilation had an optimism-corrected AUC of 0.756 and an optimism-corrected calibration slope of 0.95. Because these predictors were chosen after the primary model was fitted, these estimates are likely optimistic.

## 5. Discussion

### 5.1. Principal Findings

Among 183 individuals with class III obesity (BMI ≥ 40 kg/m^2^) undergoing term induction, half achieved vaginal delivery. Multiparity, which in this cohort means a previous vaginal birth, was the dominant predictor, with approximately seven-fold higher odds of vaginal delivery than nulliparity. Pre-pregnancy BMI was not independently associated with delivery mode within the class III obesity range.

After internal validation, the eight-predictor model had moderate discrimination (optimism-corrected AUC 0.746) and required shrinkage (optimism-corrected calibration slope 0.74). A slope of 0.74 indicates meaningful overfitting: without correction, predicted probabilities would be too extreme, specifically too high in individuals with a high predicted chance of vaginal delivery and too low in those with a low predicted chance. Shrinkage corrects this on average but does not replace external validation. Its predictors are available at admission for induction, before cervical ripening. The model has not been externally validated and is not ready for clinical use.

### 5.2. Results in the Context of What Is Known

The vaginal delivery rate of 50% aligns with data from other class III obesity cohorts. Paidas Teefey and colleagues reported a cesarean delivery rate of approximately 50% among patients with class III obesity undergoing induction, rising to 60–70% in those with BMI greater than 50 [24].

The dominant role of parity is consistent with that reported in the prior literature. Multiparous individuals have shorter labors and higher vaginal delivery rates across BMI strata [24,25,26]. Only 36.5% of nulliparous individuals in our cohort delivered vaginally, which reflects the difficulty of a first induction in class III obesity, where cesarean delivery is technically difficult and associated with higher morbidity [2,3,4].

BMI was not independently associated with delivery mode within the class III obesity range, although BMI is associated with cesarean delivery across broader BMI ranges [6,24]. This suggests that, above 40 kg/m^2^, further increases in BMI add little to the prediction once parity and maternal age are known. This differs from the larger cohort of Paidas Teefey and colleagues, in which cesarean rates rose above a BMI of 50 [24], and our sample may have been too small to detect a modest effect. Only 12 individuals had a BMI ≥ 60 kg/m^2^, so our findings cannot be extended with confidence to this group.

Existing induction prediction tools developed in mixed-BMI populations show moderate discrimination when applied to class III obesity (AUC approximately 0.75) [24]. Our model, developed within class III obesity, achieved similar discrimination after internal validation. Higher discrimination has been reported in women with obesity when intrapartum variables such as epidural use and birth weight are included [27], but such variables are not available when induction is planned. Whether a population-specific model improves on general tools can only be tested by validating both in the same external cohort.

Unlike the Bishop score, which focuses on cervical assessment [7], our model combines cervical dilation with maternal characteristics and comorbidities. We could not compare the two in this cohort.

### 5.3. Clinical Implications

The most consistent finding, and the one least dependent on the model, is that a previous vaginal birth, not BMI, determined the chance of vaginal delivery after induction. If validated, the model could be used at admission for induction, after the first cervical examination and before cervical ripening, to give individuals already planned for induction an estimate of their chance of vaginal delivery. It is not a tool for choosing between induction and planned cesarean delivery, because planned cesarean delivery was not studied. Because the unshrunken model was overfitted, predicted probabilities should not guide individual counseling until calibration is confirmed in an external cohort. The worked examples in Table 2 and the point score in Figure 1 are illustrative translations of the equation, not tools for clinical decision-making. The lack of association between BMI and delivery mode within class III obesity suggests that BMI alone should not drive counseling toward cesarean delivery when other factors are comparable.

### 5.4. Research Implications

The model is not ready for clinical use. The next step is external validation in a multicenter cohort of individuals with class III obesity undergoing induction, with at least 100 vaginal and 100 cesarean deliveries [28], assessing discrimination, calibration-in-the-large, and calibration slope, and recalibrating the intercept if needed. Prior vaginal birth, cervical dilation, and ripening method should be recorded prospectively. If performance is confirmed, implementation studies should evaluate effects on counseling outcomes (e.g., decisional conflict) and on delivery outcomes. The exploratory parsimonious model, with maternal age, parity, and cervical dilation only, performed at least as well as the eight-predictor model. Its predictors were chosen after the primary model was fitted, so its performance is likely overestimated. If it performs similarly in an external cohort, a three-predictor model would be simpler to use in practice, and both models should be validated in the same cohort.

Prior bariatric surgery had a low point estimate (aOR 0.19), but this was based on six individuals, the confidence interval was wide (0.02–1.48), and the type of procedure was not recorded. Larger cohorts are needed to study labor outcomes after bariatric surgery, a growing subgroup [29].

### 5.5. Strength and Limitations

We focused exclusively on individuals with class III obesity, a population in which counseling is difficult and which is often pooled with lower BMI categories in prior prediction models. Candidate predictors were prespecified, missing data were handled with multiple imputation, and performance was internally validated with bootstrap resampling, with shrinkage to correct for overfitting, following TRIPOD [9]. Composite maternal and neonatal morbidity were reported as descriptive information by delivery mode.

This was a single-center retrospective study, and external validation is required before implementation, as performance may vary with population and practice patterns. The sample of 183 individuals was smaller than the approximately 262 recommended for developing an eight-predictor model [23], which led to overfitting and the need for shrinkage. Cervical dilation was missing in 41 individuals (22.4%), more often in nulliparous individuals and in those who delivered by cesarean; multiple imputation reduces but does not remove the potential for bias. Bishop score components other than dilation were not consistently recorded (cervical effacement was missing in 59 individuals), so we could not compare the model with the Bishop score. The recorded indication for induction was non-specific in 30% of individuals, there were no standard criteria for failed induction, and induction practice may have changed over the 10-year study period. Labor dystocia accounted for three quarters of cesarean deliveries, and 77% of cesarean deliveries with a recorded dilation were performed before 6 cm. Thresholds for diagnosing failed induction or labor arrest differ between centers, so the predicted probability of vaginal delivery may not apply to units that use different criteria. The model applies only to individuals without a prior cesarean delivery (three were excluded). Finally, EFW was not included because timely ultrasound estimates were inconsistently available and their accuracy is reduced in obesity [15]; birth weight did not improve the model in a sensitivity analysis.

## 6. Conclusions

Among individuals with class III obesity undergoing term induction, approximately half achieved vaginal delivery. Multiparity, which in this study reflects a previous vaginal birth, was the dominant predictor, whereas BMI within the class III range was not independently associated with delivery mode. The main clinical message is as follows: within class III obesity, obstetric history is more informative than BMI when counseling about the chance of vaginal delivery after induction. This internally validated model requires external validation before it is used for counseling.

## Figures and Tables

**Table 3:** Baseline and induction characteristics by delivery mode.

Characteristic	Vaginal Delivery (*n* = 92)	Cesarean Delivery (*n* = 91)	SMD
**Maternal and pregnancy characteristics**			
Maternal age, years	31.1 ± 4.8	33.3 ± 5.3	0.42
Nulliparous	46 (50.0)	80 (87.9)	0.90
Multiparous (previous vaginal birth)	46 (50.0)	11 (12.1)	
Pre-pregnancy BMI, kg/m^2^	50.5 ± 5.3	50.5 ± 5.3	0.01
BMI 40.0–49.9	52 (56.5)	45 (49.5)	0.14
BMI 50.0–59.9	35 (38.0)	39 (42.9)	0.10
BMI ≥ 60.0	5 (5.4)	7 (7.7)	0.09
Gestational weight gain, kg	7.4 ± 18.6	10.4 ± 11.9	0.19
Prior bariatric surgery	2 (2.2)	4 (4.4)	0.12
Any hypertensive disorder	31 (33.7)	45 (49.5)	0.32
Any diabetes mellitus	27 (29.3)	24 (26.4)	0.07
Gestational diabetes	19 (20.7)	15 (16.5)	0.11
Pregestational diabetes	8 (8.7)	9 (9.9)	0.04
**Induction (descriptive)**			
GA at delivery, completed weeks	38.5 ± 1.0	38.6 ± 0.9	0.14
Cervical dilation at admission, cm ^a^	1.7 ± 1.4	1.0 ± 1.3	0.47
Indication for induction			
Hypertensive disorder	20 (21.7)	26 (28.6)	0.16
Diabetes	12 (13.0)	16 (17.6)	0.13
Post-dates	5 (5.4)	8 (8.8)	0.13
Suspected large for gestational age	6 (6.5)	7 (7.7)	0.05
Elective	9 (9.8)	6 (6.6)	0.12
Prelabor rupture of membranes	4 (4.3)	1 (1.1)	0.20
Fetal indication ^b^	5 (5.4)	3 (3.3)	0.10
Other or unknown ^c^	31 (33.7)	24 (26.4)	0.16
Cervical ripening method			
Prostaglandin alone	25 (27.2)	25 (27.5)	0.01
Balloon catheter alone	24 (26.1)	32 (35.2)	0.20
Prostaglandin and balloon catheter	11 (12.0)	23 (25.3)	0.35
None (amniotomy and/or oxytocin only)	32 (34.8)	11 (12.1)	0.56
Oxytocin used ^d^	77/79 (97.5)	70/82 (85.4)	0.44
Rupture of membranes			
Artificial	80 (87.0)	68 (74.7)	0.31
Spontaneous ^e^	11 (12.0)	11 (12.1)	0.00
Intact at cesarean or not recorded	1 (1.1)	12 (13.2)	0.48
**Neonatal**			
Birth weight, g	3410 ± 465	3462 ± 473	0.11

Data are shown as mean ± SD or n (%). Prior cesarean delivery was an exclusion criterion, so all multiparous individuals had a previous vaginal birth. Induction characteristics are descriptive and were not model predictors. BMI = body mass index; GA = gestational age; SMD = standardized mean difference. ^a^ Recorded in 142 individuals (75 vaginal; 67 cesarean). ^b^ Abnormal fetal testing, fetal growth restriction, oligohydramnios, polyhydramnios, or fetal anomaly. ^c^ Includes one individual with no recorded indication. ^d^ Recorded in 161 individuals (79 vaginal; 82 cesarean). ^e^ Includes one individual with both spontaneous and artificial rupture recorded, counted as spontaneous.

**Table 4:** Secondary outcomes by delivery mode (unadjusted, descriptive).

Outcome	Vaginal Delivery (n = 92)	Cesarean Delivery (n = 91)	SMD	Risk Difference, % (95% CI) ^b^
**Maternal**				
Composite maternal morbidity ^a^	9/92 (9.8)	13/91 (14.3)	0.14	4.5 (−5.2 to 14.3)
Chorioamnionitis or intrapartum fever	6/89 (6.7)	3/61 (4.9)	0.08	
Blood transfusion	3/91 (3.3)	2/90 (2.2)	0.07	
Postpartum infection ^c^	1/91 (1.1)	3/90 (3.3)	0.15	
Readmission within 7 days	0/91 (0.0)	7/90 (7.8)	0.41	
Estimated blood loss, mL (N)	382 ± 234 (25)	839 ± 266 (82)	1.82	
Length of postpartum stay, days (N)	1.4 ± 2.3 (66)	2.5 ± 3.8 (73)	0.35	
Shoulder dystocia ^d^	5/90 (5.6)	Not applicable		
**Neonatal**				
Composite neonatal morbidity ^e^	9/92 (9.8)	17/91 (18.7)	0.26	8.9 (−1.3 to 19.2)
Delivery room resuscitation	7/92 (7.6)	15/91 (16.5)	0.28	
NICU admission	2/87 (2.3)	3/85 (3.5)	0.07	
5 min Apgar score < 7	3/87 (3.4)	3/85 (3.5)	0.00	

Data are shown as n/N (%) or mean ± SD (N), where N is the number with the outcome recorded. Comparisons are unadjusted and descriptive; delivery mode is an outcome of labor, not an assigned exposure. CI = confidence interval; NICU = neonatal intensive care unit; SMD = standardized mean difference. ^a^ Chorioamnionitis or intrapartum fever, blood transfusion, postpartum infection (excluding mastitis), or hospital readmission within 7 days. Estimated blood loss and length of stay are not part of the composite. ^b^ Cesarean minus vaginal delivery, in percentage points (Newcombe hybrid score interval). ^c^ Endometritis after vaginal delivery; surgical site infection after cesarean delivery. ^d^ Denominator is vaginal deliveries. ^e^ Delivery room resuscitation, NICU admission, or 5 min Apgar score < 7.

## Data Availability

The data are not publicly available because of institutional privacy requirements. De-identified data may be shared upon reasonable request to the corresponding author, subject to approval by the Research Ethics Board and a data sharing agreement.

## Supplementary Materials

The following supporting information can be downloaded at https://www.mdpi.com/article/10.3390/jcm15218371/s1, Table S1. TRIPOD checklist for prediction model development (Type 1b: development with internal validation by resampling). Table S2. Characteristics of individuals with and without recorded cervical dilation at admission. Table S3. Model performance of the 8-predictor model. Table S4. Sensitivity analyses. Figure S1. Secondary outcomes by delivery mode.
