# Supplementary material for: Prediction of Vaginal Delivery in Pregnant Individuals with Class III Obesity Undergoing Induction of Labor: Development and Internal Validation of a Prediction Model
Source: J Clin Med. 2026 Oct 27;15(21):8371. doi: 10.3390/jcm15218371 (PMC13763801; doi:10.3390/jcm15218371)
Supplement: Supplementary file 1 [file jcm-15-08371-s001.zip › jcm-4530080-supplementary.pdf]

Supplementary Materials

Prediction of Vaginal Delivery in Pregnant Individuals with Class III Obesity Undergoing Induction of Labor: A Clinical Prediction Model

Table S1. TRIPOD checklist for prediction model development (Type 1b: development with internal validation by resampling).

Section	Item	Checklist item	Location
Title	1	Identify the study as developing a prediction model, the target population, and the outcome	Title
Abstract	2	Summary of objectives, design, setting, participants, sample size, predictors, outcome, analysis, results, and conclusions	Abstract
Background	3a	Medical context and rationale	Section 1
Objectives	3b	Objectives, including development and validation	Section 1, final paragraph
Source of data	4a	Study design and source of data	Section 2.1
	4b	Key study dates	Section 2.1
Participants	5a	Study setting	Section 2.1
	5b	Eligibility criteria	Section 2.2
	5c	Details of treatments received (induction methods)	Section 2.2; Table 1
Outcome	6a	Definition of the outcome and how it was assessed	Section 2.4
Predictors	7a	Definition of all predictors and when they were measured	Section 2.3
Sample size	8	How the study size was arrived at	Section 2.10
Missing data	9	How missing data were handled	Section 2.5
Statistical analysis	10a	How predictors were handled	Sections 2.3 and 2.6
	10b	Type of model, model-building procedure, and internal validation	Sections 2.6 and 2.7
	10d	Measures used to assess model performance	Sections 2.7 and 2.8
Risk groups	11	Details of any risk groups created	Section 4.3
Participants	13a	Flow of participants, numbers with and without the outcome	Section 3.1
	13b	Characteristics of participants, including missing data	Table 1; Section 3.1; Table S2
Model development	14a	Number of participants and outcome events in each analysis	Sections 3.1 and 4.6
	14b	Unadjusted associations between candidate predictors and outcome	Table 1
Model specification	15a	Full prediction model (all coefficients and intercept)	Table 3; Section 4.5
	15b	How to use the prediction model	Section 4.5; Table 4; Figure 4
Model performance	16	Performance measures with confidence intervals	Section 4.2; Figure 2; Table S3
Discussion	18	Limitations	Section 5.5
	19b	Overall interpretation	Section 5
	20	Potential clinical use and implications for research	Sections 5.3 and 5.4

Other information	21	Supplementary resources	Supplementary Materials
	22	Source of funding	Funding statement

Table S2. Characteristics of individuals with and without recorded cervical dilation at admission.

Characteristic	Recorded (n=142)	Missing (n=41)	SMD
Maternal age, years	32.2 ± 5.4	32.2 ± 4.3	0.01
Nulliparous	93/142 (65.5)	33/41 (80.5)	0.34
Pre-pregnancy BMI, kg/m²	50.4 ± 5.4	50.9 ± 4.7	0.10
Gestational weight gain, kg [a]	8.5 ± 14.3	10.3 ± 19.8	0.10
Any hypertensive disorder	61/142 (43.0)	15/41 (36.6)	0.13
Any diabetes mellitus	42/142 (29.6)	9/41 (22.0)	0.18
Prior bariatric surgery	5/142 (3.5)	1/41 (2.4)	0.06
Vaginal delivery	75/142 (52.8)	17/41 (41.5)	0.23

Data are mean ± SD or n/N (%). a Recorded in 141 and 40 individuals, respectively. The complete-case analysis included the 141 individuals with recorded cervical dilation and gestational weight gain. SMD = standardized mean difference.

Table S3. Model performance of the 8-predictor model.

Measure	Apparent	Optimism	Optimism-corrected
AUC (95% CI)	0.784 (0.713–0.844)	0.038	0.746 (0.675–0.806)
Brier score	0.190	−0.021	0.211
Calibration intercept	0.000 [a]	−0.005	0.005
Calibration slope	1.000 [a]	0.264	0.736

Apparent performance and optimism were averaged across 30 imputed datasets with 200 bootstrap samples each. a The apparent calibration intercept and slope are 0 and 1 by definition when a logistic model is evaluated in its development data. Uniform shrinkage factor applied to the published equation: 0.74. Cox–Snell R^2 0.24; Nagelkerke R^2 0.32. Minimum sample size by Riley et al. criterion (i), expected shrinkage ≤10%: 262.

Classification by the shrunken model at three predicted probabilities of vaginal delivery (n=183).

Predicted probability ≥	Predicted vaginal delivery, n	TP	FP	FN	TN	Sensitivity, %	Specificity, %	PPV, %	NPV, %
30%	154	89	65	3	26	96.7	28.6	57.8	89.7
50%	78	59	19	33	72	64.1	79.1	75.6	68.6
70%	45	37	8	55	83	40.2	91.2	82.2	60.1

Predicted probabilities were averaged across imputed datasets for each individual. These are descriptive categories, not thresholds for recommending cesarean delivery. FN = false negative; FP = false positive; NPV = negative predictive value; PPV = positive predictive value; TN = true negative; TP = true positive.

Table S4. Sensitivity analyses.

	Primary model (multiple imputation, n=183)	Complete-case (n=141)	Primary model + birth weight (n=183)	Parsimonious model [a] (n=183)
Apparent AUC	0.784	0.781	0.788	0.765
Optimism-corrected AUC	0.746	Not estimated	0.746	0.756
Optimism-corrected calibration slope	0.74	Not estimated	0.72	0.95
Events per variable	11.5	9.3	10.2	30.7
**aOR (95% CI)**				
Maternal age, per year	0.91 (0.85–0.98)	0.94 (0.87–1.01)	0.91 (0.84–0.97)	0.91 (0.85–0.98)
Multiparous	7.38 (3.26–16.70)	6.64 (2.69–16.39)	7.57 (3.30–17.33)	6.94 (3.16–15.22)
BMI, per kg/m²	0.99 (0.93–1.06)	1.02 (0.95–1.10)	0.99 (0.93–1.06)	Not included
Gestational weight gain, per kg	0.99 (0.96–1.01)	1.00 (0.97–1.03)	0.99 (0.97–1.02)	Not included
Prior bariatric surgery	0.19 (0.02–1.48)	0.19 (0.02–1.63)	0.20 (0.03–1.53)	Not included
Cervical dilation, per cm	1.23 (0.91–1.66)	1.27 (0.93–1.73)	1.27 (0.92–1.76)	1.22 (0.92–1.62)
Hypertensive disorder	0.62 (0.31–1.25)	0.44 (0.20–0.98)	0.53 (0.25–1.10)	Not included
Diabetes mellitus	1.32 (0.59–2.95)	1.30 (0.51–3.30)	1.49 (0.65–3.43)	Not included
Birth weight, per 100 g	Not included	Not included	0.93 (0.86–1.02)	Not included

a Maternal age, parity, and cervical dilation; these predictors were chosen after the primary model was fitted, so its optimism-corrected estimates are likely optimistic. The birth weight model used a separate imputation that included birth weight (one value missing). Optimism correction was not performed for the complete-case analysis. aOR = adjusted odds ratio; AUC = area under the receiver operating characteristic curve; CI = confidence interval.

Figure S1. Secondary outcomes by delivery mode.

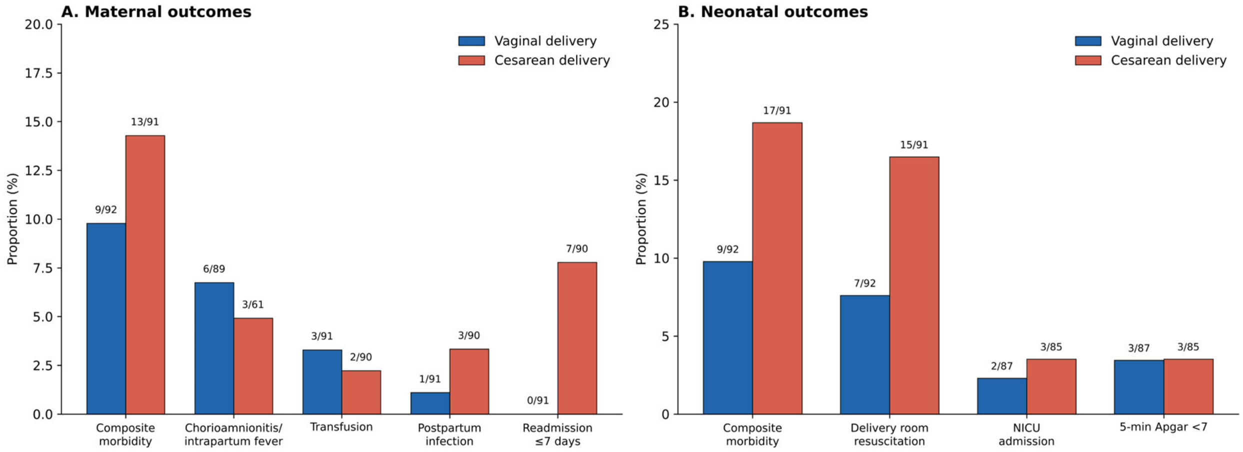

(A) Maternal and (B) neonatal outcomes. Bars show the percentage with the outcome; numerators and denominators (individuals with the outcome recorded) are shown above each bar. Comparisons are unadjusted and descriptive; delivery mode is an outcome of labor, not an assigned exposure.
